# Unsupervised dietary-behavioral profiling identifies PFAS (per- and polyfluoroalkyl substances) and metal exposure patterns in Korean adults

**DOI:** 10.1038/s41598-026-64855-4

**Published:** 2026-08-04

**Authors:** SangHee Hong, JeongBeom Lee, Hak-Jae Kim

**Affiliations:** 1 Department of Medical Sciences, Graduate School, Soonchunhyang University, Asan, Republic of Korea; 2 Department of Physiology, College of Medicine, Soonchunhyang University, Cheonan, 31151 Republic of Korea; 3 Advanced Pharmacology and Physiology Integrated Sciences (AXIS), College of Medicine, Soonchunhyang University, Cheonan, 31151 Republic of Korea; 4 Department of Pharmacology, College of Medicine, Soonchunhyang University, Cheonan, 31151 Republic of Korea

**Keywords:** Nutritional epidemiology, Unsupervised learning, Dietary behavior patterns, Environmental exposure biomarkers, Korea National Environmental Health Survey (KoNEHS), Biomarkers, Diseases, Environmental sciences, Health care, Medical research, Risk factors

## Abstract

Beyond “what and how much you eat,” modern nutritional epidemiology is paying attention to how dietary and behavioral patterns, which combine food selection and the context of cooking, packaging, and ingestion, organize health and environmental exposure. This cross-sectional secondary analysis used adult data from the 4th Korea National Environmental Health Survey (KoNEHS Cycle 4, 2018–2020; *n* = 4239). Multi-question variables related to diet, cooking, packaging, and ingestion behaviors were mapped into a latent space through unsupervised learning, clusters were derived, and the exposure levels of PFAS, bisphenols, urinary cadmium, and blood lead biomarkers were compared by cluster. As a result, serum PFAS concentrations were higher in the “basic diet/seafood propensity” cluster, with weighted geometric means ranging from 10.998 to 21.767 µg/L for PFOS and from 5.082 to 8.323 µg/L for PFOA across clusters. In the “packaging/contact propensity” cluster, higher PFOA was observed in the adjusted analysis, while blood lead showed an elevated direction but was not robust after FDR correction. On the other hand, the differences between clusters for bisphenols and urinary cadmium were not robust when considering covariates and multiple comparisons, suggesting the possibility that the variability of single urine measurements, uncertainty in exposure assessment, and complexity of indicator characteristics may have affected the results. This study provides an empirical basis for identifying population-level dietary-behavioral exposure profiles and informing targeted risk communication by showing how lifestyle-based dietary and behavioral structures are associated with environmental exposure patterns before directly linking dietary patterns with diseases.

## Introduction

Modern nutritional epidemiology is extending its interest beyond “what and how much people eat” to how behavioral patterns, which combine food selection and the context of cooking, packaging, and ingestion, organize health and exposure^1^. This shift in perspective, coupled with the cumulative evidence that dietary patterns are linked to disease prevention and physiological mechanisms, has developed in the direction of supplementing the limitations of single nutrient-centered interpretation^2^. In fact, it has been emphasized that diet should be understood as a multi-layered determinant linked to society, policy, and environment beyond individual choice^3^. At the same time, it has been repeatedly suggested that measurement errors and residual disturbances in questionnaire-based dietary data can distort estimates^4^.

In this context, diet and behavior can mediate major exposure pathways for environmentally hazardous substances, and in particular, Per- and Polyfluoroalkyl Substances (PFAS) can flow through food and drinking water pathways and have a long residual in the body, resulting in a group-level burden^5^. Epidemiologic and toxicological discussions on the health risks of PFAS are constantly being updated, including newly reported risk signals, supporting the public health need for exposure reduction^6^. PFAS exposure through food may vary depending on food culture, food supply chain, and cooking practices, requiring an evaluation that considers heterogeneity within a population group^7^. In addition, the report that predictors of blood concentration may vary as individual characteristics (age, gender, socioeconomic location) and lifestyle work together suggests that exposure inequality may occur even within the same country^8^.

Bisphenols are a representative group of substances associated with food contact materials (plastics, epoxy resins, etc.), and it has been summarized that daily exposure can occur extensively^9^. In particular, randomized cross-examination results indicating that canned intake is associated with a short-term increase in urine Bisphenol A concentration show that the packaging form can rapidly change exposure^10^. Furthermore, studies on estimating the amount of transition in canned beverages and canned foods reinforce that “what container and packaging” determines exposure as well as “what do you eat”^11^. Even in the case of metal exposure, cadmium has been discussed as an important axis of public health implications for chronic environmental exposure and food contribution^12^. Lead has been consistently evoking the priorities of exposure reduction, with the evidence that effects can be observed in vulnerable groups even at low levels of environmental exposure^13^. In addition, the cadmium risk assessment among foods suggests the direction that high-exposure groups should be identified in the general population and long-term risk management should be performed^14^.

In connection with exposure to each of these harmful factors, research dealing with both diet and environmental exposure is also linked to discussions on sustainability and planetary health, and reinforces the view that the food system is intertwined with environmental pollution and human exposure^15^. In order to deal with this complexity, an approach has recently spread to project high-dimensional dietary and behavior data into latent spaces using unsupervised learning and dimensional reduction and to summarize subgroups with similar behavioral combinations into clusters^16^. Clustering can go beyond simple average comparisons and characterize population-level exposure-related patterns, providing practical clues to policy targeting and risk communication^17^. Recent studies are moving beyond pre-assumed dietary scores or single variable-centered modeling to explore potential structures of diet and behavior based on patterns shown by observed data themselves and to find out how the structures organize group differences in environmental exposure or health indicators^2,4^. Unsupervised learning is useful at this point: by mapping the multidimensional dietary patterns inherent in the data rather than fitting them to predetermined classifications by researchers, it can specifically reveal subgroups (e.g., those with similar basic dietary intake but significantly different packaging and contact behaviors) that are easy to miss in conventional approaches^16,17^. Furthermore, understanding dietary patterns as a continuous spectrum in latent space rather than simplifying them into “several categories” may better reflect the reality that exposure vulnerability does not suddenly change at “boundary” but gradually shifts with lifestyle changes^16^.

Clusters derived from latent spaces can be interpreted as behavior-based profiles summarizing dietary and behavioral combinations of real life, not just statistical classification results^2,15^, which is highly likely to be used for public health. For example, if a pattern in which seafood consumption is prominent appears in combination with a specific age, sex, or lifestyle, or if packaging and contact-related behaviors appear in combination with the use of super-processed foods, this answers the question more directly^6,7^. In other words, unsupervised learning-based dietary pattern analysis is significant in that it provides targeted risk communication clues by indicating which behavioral combinations may be relevant to exposure differences beyond simple association reports^3^.

On the other hand, in order to present unsupervised learning results as a practical basis, it is necessary to check whether the cluster is not excessively random in the latent space and has a certain level of separation and consistency^18,19^. However, the core of this study is not to assert the excellence of specific indicators themselves, but to visualize the latent structure of diet and nutrition-related behavior and to describe and compare at the population level whether the structure is linked to group differences in environmentally harmful substance biomarkers^1,21^. In addition, since national survey data, like KoNEHS, should be analyzed with attention to the sample design, it is important to support the representativeness and interpretability of the results through summaries and comparisons that consider survey weights^20–23^. As a result, this study aims to connect the methodological expansion of nutritional epidemiology to the public health applicability by structuring diet and behavior patterns based on data through unsupervised learning and presenting differences between the patterns and biomarkers of major environmentally harmful substances^1,2^.

## Methods

### Study design and secondary data source

This cross-sectional secondary analysis used adult raw data from the Korean National Environmental Health Survey (KoNEHS) Cycle 4 (2018–2020). A total of 4,239 adults were included in the analysis. KoNEHS was conducted using a complex sampling design to support national representativeness, and the raw data are provided with sample design weights. The present analysis used de-identified secondary data including demographic variables, diet- and food-related behavioral questionnaire items, urinary cotinine, urinary creatinine, survey weights, and biomarker concentrations of bisphenols, PFAS, urinary cadmium, and blood lead. The aim of this study was not to verify causal associations between specific nutrients and disease outcomes, but to map the latent structure of diet- and nutrition-related behaviors at the population level using unsupervised learning and to compare environmental chemical biomarker concentrations across the derived dietary-behavioral clusters.

### Dietary-behavioral variable construction

Rather than estimating precise nutrient intake, the KoNEHS dietary items reflect usual intake behaviors and the relative intensity of diet-related behaviors. In this study, these items were regarded as proxy indicators of dietary and food-related behaviors. Instead of interpreting individual items separately, we defined multi-item dietary-behavioral domains as the main unit of analysis. To improve interpretability, diet- and food-related variables were organized into the following five domains:

(1) ultra-processed and processed food consumption, including cup noodles, fast food or chicken products, instant or packaged foods, and canned foods; (2) basic food group intake, including grains, vegetables, fruits, kimchi, beans, processed bean products, and dairy products; (3) seafood intake, including fish, crustaceans, shellfish, and seaweed; (4) high-temperature cooking, including the frequency of grilled or roasted foods; and (5) food packaging and container-contact behaviors, including the use of plastic containers for hot food or water, use of coated cookware, and consumption of packaged or delivered foods.

This domain structure was designed to represent dietary behavior as a continuous spectrum that reflects not only what and how often individuals consume foods, but also how behavioral characteristics are combined during intake, cooking, packaging, and food-container contact.

### Frequency-based rescaling and standardization

The multiple dietary items of KoNEHS are measured by ordered categories of “almost no eating–monthly–weekly–daily.” Since Uniform Manifold Approximation and Projection (UMAP) preserves neighborhood structures based on distance, the interval between categories may not sufficiently reflect the actual intake intensity if the category code values are used as they are. Therefore, in this study, frequency-based rescaling was applied to ordinal variables indicating the frequency of intake or use.

Specifically, for the frequency of intake and use variables, each category was converted to an approximation of “number of intake or use events per year” (e.g., once a month = 12 times/year, once a week = 52 times/year, once a day = 365 times/year). Interval categories (e.g., 2–3 times a week) were converted to median-based approximations. On the other hand, since the cooking method variable reflects the order of cooking characteristics rather than frequency, the ordinal information was maintained without applying frequency conversion. After that, all input variables were standardized as z-scores to correct scale differences between variables.

### UMAP-based dimension reduction and K-means clustering

By applying UMAP to the standardized dietary variable matrix, individual dietary patterns were projected into a two-dimensional latent space. UMAP preserves the local neighboring structure of high-dimensional data and can express nonlinear structures, making it suitable for dietary behavior data that are expected to be multidimensional and nonlinear.

Based on the two-dimensional coordinates (UMAP1, UMAP2) calculated by UMAP, K-means cluster analysis was performed to classify individuals with similar dietary structures into clusters. The number of clusters (k) was determined by comprehensively considering (1) indicators reflecting the degree of cohesion and separation between clusters, (2) the interpretability of the dietary profile for each cluster, and (3) the occurrence of excessively unbalanced clusters, and k = 5 was finally adopted in this study. UMAP was implemented with fixed random seeds to improve reproducibility under the same data and software conditions. Demographic variables and concentrations of environmental hazardous substances were not used as inputs for UMAP embedding and cluster derivation.

### Biomarker definition, measurement units, and statistical comparison

The biomarkers compared in this study were urinary bisphenol A (BPA), urinary bisphenol F (BPF), urinary bisphenol S (BPS), serum perfluorooctane sulfonate (PFOS), serum perfluorooctanoic acid (PFOA), urinary cadmium (Ucd), and blood lead (BPb). Chemical biomarker concentrations were obtained from the KoNEHS laboratory biomonitoring data. The original measurement units were µg/L for urinary BPA, BPF, BPS, cotinine, and cadmium; µg/L for serum PFOS and PFOA; µg/dL for blood lead; and g/L for urinary creatinine. In consideration of the right-skewed concentration distributions, natural logarithmic transformation was applied to all biomarker concentrations. Regression and mean estimation were performed on the natural log scale, and the results were presented after converting to the original concentration scale through exponential transformation.

The unadjusted exposure level for each cluster was presented as a weighted geometric mean (GM) and a 95% confidence interval by applying the survey weight. For adjusted comparisons between clusters, the adjusted geometric mean ratio (GMR) was calculated using a weighted least squares (WLS) regression model with natural log-transformed concentration as the dependent variable. The adjustment model included age, sex, and urinary cotinine as covariates. For urinary cadmium, urinary creatinine (U_crea) was additionally included to account for urinary dilution. Considering multiple comparisons due to multiple biomarkers and cluster comparisons, the p-value was corrected using the false discovery rate (FDR) according to the Benjamini–Hochberg procedure.

### Survey weights and statistical processing

In order to reflect the sampling structure of KoNEHS data, survey weights were applied in descriptive statistics and exposure comparison analyses. Sampling design weights provided by KoNEHS were used for descriptive statistics, weighted geometric mean estimation, and weighted regression-based comparisons. The weights are calculated by reflecting the sampling rate, non-response adjustment, and benchmarking adjustment, and a trimming procedure is included to mitigate the effects of extreme values. The 95% confidence interval of the regression-based comparison was calculated using robust standard errors. Samples included in the final regression analysis may be partially different for each biomarker depending on whether the exposure indicator and covariates were missing.

Data preprocessing, dimension reduction using UMAP, cluster analysis using K-means, statistical modeling, and visualization were performed in a Python-based analysis environment. The random number seed was fixed to improve reproducibility of the embedding and cluster results under the same data and software conditions.

## Results

### Cohort and cluster composition

The number of K-means clusters (k) was explored from 2 to 10 in the UMAP latent space. Silhouette and Davies–Bouldin indices were compared together, and solutions that produced excessively small or highly imbalanced clusters were avoided. Based on the balance of separation, cluster quality, and interpretability, k = 5 was selected for the final analysis. K-means clustering with k = 5 in the UMAP-based latent space included 4,239 participants and yielded five clusters: Cluster 0 (*n* = 916), Cluster 1 (*n* = 766), Cluster 2 (*n* = 892), Cluster 3 (*n* = 1,009), and Cluster 4 (*n* = 656).

The demographic and basic characteristics differed by cluster (Table 1). The average age was highest in Cluster 1 (61.1 years), followed by Cluster 3 (57.6 years), Cluster 2 (52.1 years), Cluster 0 (46.7 years), and Cluster 4 (40.0 years). The proportion of women was relatively high in Cluster 3 (63.4%) and Cluster 0 (61.1%) and lowest in Cluster 4 (42.8%). The mean value of cotinine used as an indicator of smoking/nicotine exposure was the highest in Cluster 4 (327) and the lowest in Cluster 3 (124), suggesting the possibility that lifestyle habits or exposure patterns by cluster may be heterogeneous.

**Table 1:** Participant and biomarker characteristics by dietary-behavioral cluster (k = 5).

Cluster	n	Age (years)	Female (%)	Ucd (µg/L)	BPb (µg/dL)	BPA (µg/L)	BPF (µg/L)	BPS (µg/L)	PFOS (µg/L)	PFOA (µg/L)	COT (µg/L)	U_crea (g/L)
0	916	46.7 ± 14.9	61.1	0.611 ± 0.736	1.546 ± 0.693	2.264 ± 4.025	0.839 ± 4.224	0.759 ± 2.840	16.619 ± 11.895	7.053 ± 4.872	218.57 ± 539.576	1.100 ± 0.656
1	766	61.1 ± 11.3	55.9	0.873 ± 1.226	1.881 ± 0.900	2.218 ± 4.486	0.491 ± 1.642	0.433 ± 2.092	25.983 ± 15.332	9.831 ± 5.410	167.954 ± 459.158	0.908 ± 0.557
2	892	52.1 ± 13.4	49.4	0.677 ± 0.693	1.855 ± 1.251	2.487 ± 6.462	0.488 ± 1.603	0.445 ± 1.290	18.950 ± 10.946	8.035 ± 4.239	239.153 ± 564.225	0.932 ± 0.598
3	1009	57.6 ± 13.2	63.4	0.788 ± 0.876	1.804 ± 1.018	2.065 ± 5.993	0.841 ± 6.587	0.519 ± 3.092	21.515 ± 20.746	8.629 ± 5.531	124.202 ± 387.986	0.907 ± 0.589
4	656	40.0 ± 12.8	42.8	0.499 ± 0.589	1.580 ± 0.719	3.096 ± 8.549	0.902 ± 5.785	0.578 ± 1.333	13.848 ± 9.080	6.444 ± 3.746	327.377 ± 621.436	1.164 ± 0.757

Table 1 summarizes the demographic characteristics and biomarker concentrations of the study participants according to dietary-behavioral clusters identified using UMAP-based K-means clustering (k = 5). Cluster n values are unweighted counts. Continuous variables are presented as mean ± standard deviation, and female sex is presented as percentage. Biomarker concentrations are shown on the original measurement scale for descriptive purposes. Ucd, BPA, BPF, BPS, and COT are urinary concentrations in µg/L; BPb is blood lead in µg/dL; PFOS and PFOA are serum concentrations in µg/L; and U_crea is urinary creatinine in g/L. Ucd: urinary cadmium concentration; BPb: blood lead concentration; BPA: bisphenol A; BPF: bisphenol F; BPS: bisphenol S; PFOS: perfluorooctane sulfonate; PFOA: perfluorooctanoic acid; COT: cotinine; U_crea: urinary creatinine.

**Fig. 1:**
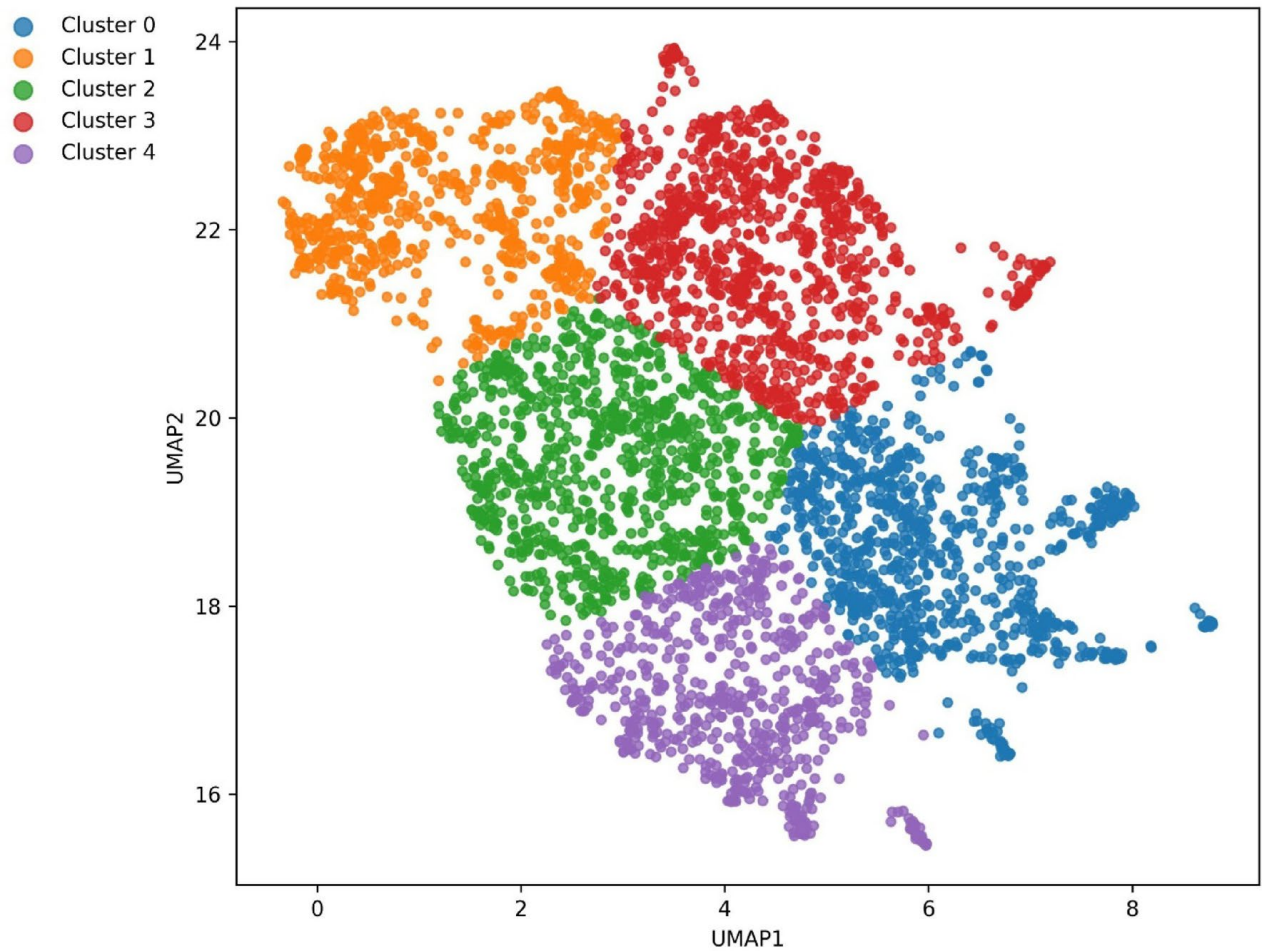
UMAP-based clustering of dietary-behavioral profiles (k = 5). K-means clustering results (k = 5) based on standardized diet- and food-related variables are visualized in a two-dimensional UMAP (Uniform Manifold Approximation and Projection) space. Each point represents an individual participant, and colors indicate cluster membership. This figure is intended to visualize the latent structure of dietary-behavioral profiles and the relative separation between clusters; the absolute distances between points should not be interpreted as quantitative dietary distances.

In UMAP visualization, the five clusters showed relatively clear spatial separation (Fig. 1), supporting that the derived clusters captured distinct dietary-behavioral patterns rather than simple average differences.

### Cluster-wise dietary domain profiles

**Fig. 2:**
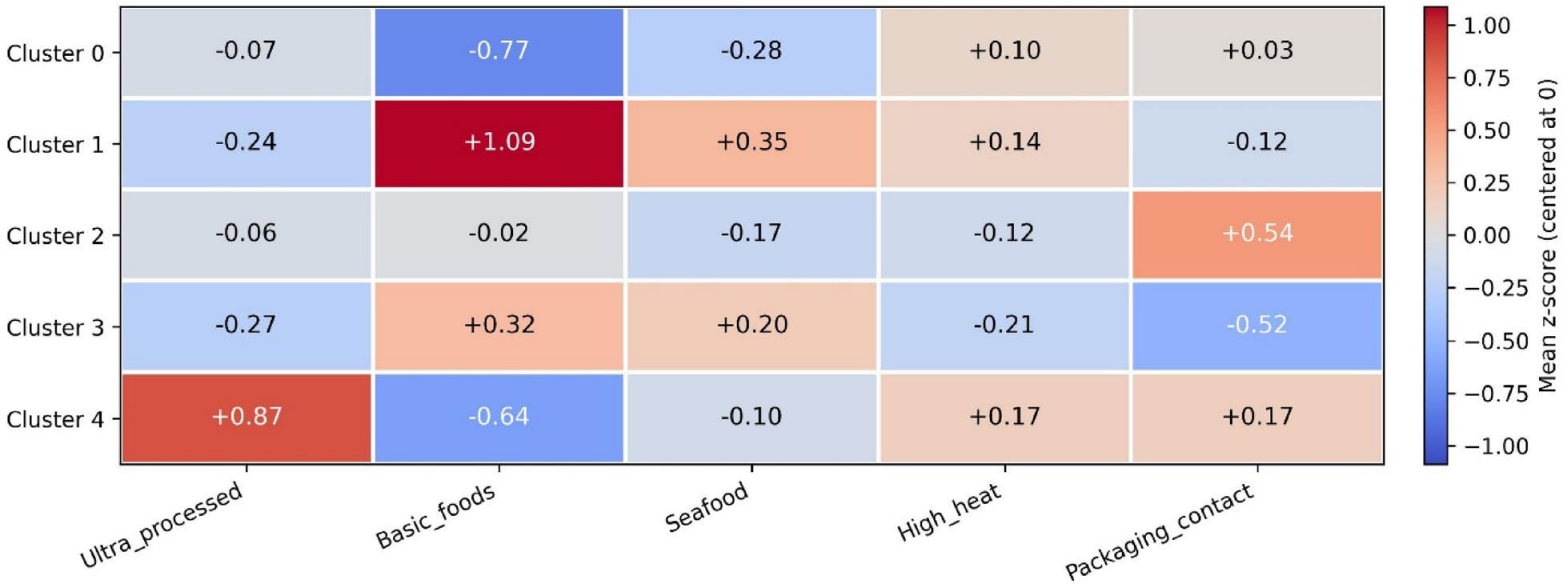
Cluster-wise dietary domain profiles. The heatmap shows the mean scores of diet- and food-related behavioral domains for each cluster as centered z-scores. Rows represent clusters, and columns represent the ultra-processed food, basic food, seafood, high-temperature cooking, and packaging/container-contact domains. Positive values indicate domain scores above the overall sample mean, whereas negative values indicate scores below the overall sample mean. Ultra_processed: ultra-processed food consumption domain; Basic_foods: basic and traditional food consumption domain; Seafood: seafood consumption domain; High_heat: high-temperature cooking domain; Packaging_contact: food packaging and container-contact domain.

Dietary-behavioral domain scores, expressed as centered z-score means, differed across clusters (Table 2; Fig. 2). Cluster 1 had the highest Basic_foods score (+ 1.09), with a positive Seafood score (+ 0.35), indicating a basic-food and seafood-oriented profile. In contrast, Cluster 0 had the lowest Basic_foods score (-0.77), representing a relatively low basic-food profile and serving as the reference group in subsequent comparisons. Cluster 4 had the highest Ultra_processed score (+ 0.87), indicating an ultra-processed food-oriented profile. Cluster 2 showed a relatively high Packaging_contact score (+ 0.54), suggesting a packaging/contact-oriented profile. Cluster 3 had positive Basic_foods (+ 0.32) and Seafood (+ 0.20) scores but a negative Packaging_contact score (-0.52), indicating a basic-food and seafood-oriented profile with relatively low packaging/contact-related behaviors. The High_heat domain showed only modest variation across clusters, ranging approximately from − 0.21 to + 0.17.

**Table 2:** Cluster-wise dietary-behavioral domain profiles (centered z-scores).

Cluster	Ultra_processed (z mean ± SD)	Basic_foods (z mean ± SD)	Seafood (z mean ± SD)	High_heat (z mean ± SD)	Packaging_contact (z mean ± SD)
0	−0.07 ± 0.86	−0.77 ± 0.81	−0.28 ± 0.71	0.10 ± 1.13	0.03 ± 1.16
1	−0.24 ± 0.80	1.09 ± 0.73	0.35 ± 1.08	0.14 ± 1.21	−0.12 ± 0.87
2	−0.06 ± 0.88	−0.02 ± 0.73	−0.17 ± 0.70	−0.12 ± 0.74	0.54 ± 0.89
3	−0.27 ± 0.84	0.32 ± 0.69	0.20 ± 1.30	−0.21 ± 0.88	−0.52 ± 0.72
4	0.87 ± 1.26	−0.64 ± 0.83	−0.10 ± 0.88	0.17 ± 0.94	0.17 ± 0.98

Table 2 presents the cluster-wise profiles of dietary and food-related behavioral domains derived from questionnaire-based items. Values represent cluster-specific mean z-scores centered at zero across the total study population, allowing comparison of the relative prominence of each domain across clusters. Positive values indicate higher-than-average scores in the corresponding domain, whereas negative values indicate lower-than-average scores. Ultra_processed: ultra-processed food consumption domain; Basic_foods: basic and traditional food consumption domain; Seafood: seafood consumption domain; High_heat: high-temperature cooking domain; Packaging_contact: food packaging and container-contact domain.

### Unadjusted exposure profiles by clusters (weighted GM with 95% CI)

**Fig. 3:**
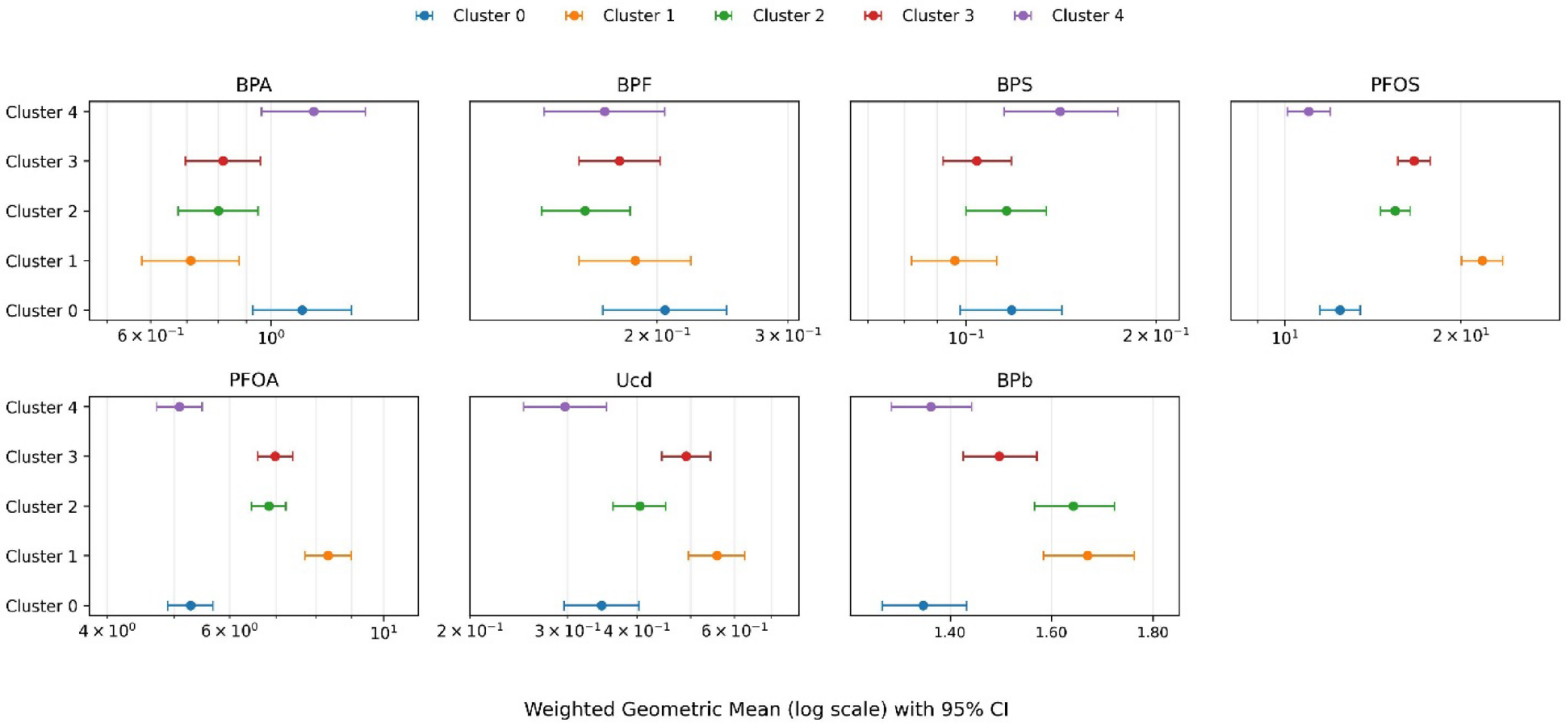
Weighted geometric mean exposure profiles by cluster. Weighted geometric means (GMs) and 95% confidence intervals of chemical biomarker concentrations are presented by dietary-behavioral cluster. Biomarker concentrations were estimated after natural logarithmic transformation and back-transformed to the original scale for presentation. Survey weights from the Korean National Environmental Health Survey (KoNEHS) were applied. This figure presents unadjusted descriptive comparisons and visually summarizes relative exposure distributions across clusters. BPA, BPF, BPS, and Ucd are urinary concentrations in µg/L; PFOS and PFOA are serum concentrations in µg/L; BPb is blood lead in µg/dL. BPA: bisphenol A; BPF: bisphenol F; BPS: bisphenol S; PFOS: perfluorooctane sulfonate; PFOA: perfluorooctanoic acid; Ucd: urinary cadmium concentration; BPb: blood lead concentration.

Weighted geometric means (GMs) and 95% confidence intervals showed descriptive exposure patterns across clusters by chemical group (Table 3; Fig. 3). For PFAS, the weighted GM of PFOS was highest in Cluster 1 (21.767 µg/L), followed by Cluster 3 (16.634 µg/L), Cluster 2 (15.450 µg/L), Cluster 0 (12.440 µg/L), and Cluster 4 (10.998 µg/L). For PFOA, Cluster 1 also showed the highest weighted GM (8.323 µg/L), followed by Cluster 3 (6.986 µg/L), Cluster 2 (6.839 µg/L), Cluster 0 (5.271 µg/L), and Cluster 4 (5.082 µg/L). For bisphenols, BPA and BPS were relatively high in Cluster 4 (BPA, 1.199 µg/L; BPS, 0.141 µg/L) and relatively low in Cluster 1 (BPA, 0.712 µg/L; BPS, 0.096 µg/L). BPF showed relatively small differences across clusters (Table 3). For metals, blood lead (BPb) tended to be higher in Cluster 1 (1.671 µg/dL) and Cluster 2 (1.643 µg/dL) and lower in Cluster 0 (1.347 µg/dL), whereas urinary cadmium (Ucd) tended to be higher in Cluster 1 (0.558 µg/L) and Cluster 3 (0.491 µg/L) and lower in Cluster 4 (0.297 µg/L). These weighted GM-based results showed descriptive differences in exposure levels across clusters. However, because covariate distributions such as age, sex, and urinary cotinine differed by cluster (Table 1), adjusted analyses were performed to examine whether these patterns were maintained after covariate adjustment. For urinary cadmium, urinary creatinine was additionally considered to account for urinary dilution.

**Table 3:** Weighted geometric means and 95% confidence intervals of chemical biomarkers by dietary-behavioral cluster.

Cluster	BPA GM (95% CI)	BPF GM (95% CI)	BPS GM (95% CI)	PFOS GM (95% CI)	PFOA GM (95% CI)	Ucd GM (95% CI)	BPb GM (95% CI)
0	1.142 (0.907, 1.437)	0.205 (0.169, 0.247)	0.118 (0.098, 0.143)	12.440 (11.489, 13.469)	5.271 (4.895, 5.676)	0.346 (0.288, 0.414)	1.347 (1.270, 1.430)
1	0.712 (0.567, 0.893)	0.187 (0.155, 0.225)	0.096 (0.082, 0.112)	21.767 (19.777, 23.956)	8.323 (7.627, 9.083)	0.558 (0.488, 0.638)	1.671 (1.590, 1.756)
2	0.800 (0.675, 0.948)	0.160 (0.139, 0.185)	0.116 (0.099, 0.135)	15.450 (14.541, 16.417)	6.839 (6.441, 7.261)	0.405 (0.358, 0.457)	1.643 (1.559, 1.732)
3	0.816 (0.697, 0.955)	0.178 (0.156, 0.203)	0.104 (0.093, 0.118)	16.634 (15.454, 17.903)	6.986 (6.556, 7.445)	0.491 (0.441, 0.547)	1.497 (1.423, 1.574)
4	1.199 (0.969, 1.483)	0.170 (0.142, 0.203)	0.141 (0.115, 0.174)	10.998 (10.043, 12.044)	5.082 (4.691, 5.505)	0.297 (0.244, 0.362)	1.362 (1.288, 1.439)

Table 3 presents the weighted geometric means (GMs) and 95% confidence intervals (CIs) of chemical biomarker concentrations across dietary-behavioral clusters identified using UMAP-based K-means clustering. Estimates were calculated on the natural log-transformed scale and back-transformed to the original scale for presentation. Survey sampling weights were applied for descriptive comparison across clusters. BPA, BPF, BPS, and Ucd are urinary concentrations in µg/L; PFOS and PFOA are serum concentrations in µg/L; BPb is blood lead in µg/dL. BPA: bisphenol A; BPF: bisphenol F; BPS: bisphenol S; PFOS: perfluorooctane sulfonate; PFOA: perfluorooctanoic acid; Ucd: urinary cadmium concentration; BPb: blood lead concentration.

### Adjusted exposure differences (WLS-based adjusted GMR)

**Fig. 4:**
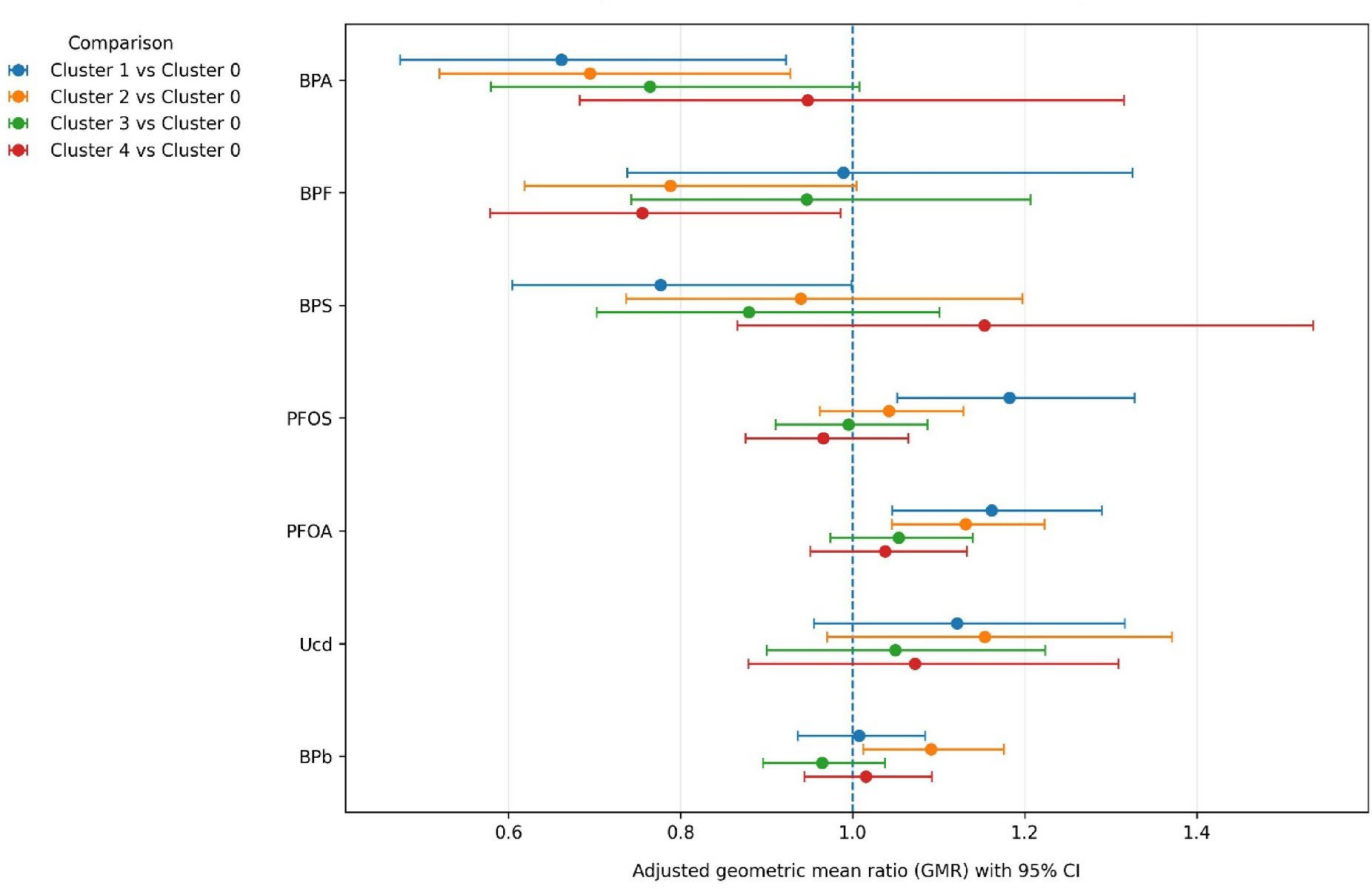
Adjusted geometric mean ratios of chemical biomarkers by cluster. Adjusted geometric mean ratios (GMRs) and 95% confidence intervals comparing each cluster with Cluster 0 as the reference are presented as forest plots. Estimates were calculated using weighted least squares regression models with natural log-transformed biomarker concentrations as dependent variables. Models were adjusted for age, sex, and urinary cotinine as an indicator of smoking or nicotine exposure. For urinary cadmium, urinary creatinine was additionally included to account for urinary dilution. BPA: bisphenol A; BPF: bisphenol F; BPS: bisphenol S; PFOS: perfluorooctane sulfonate; PFOA: perfluorooctanoic acid; Ucd: urinary cadmium concentration; BPb: blood lead concentration.

**Fig. 5:**
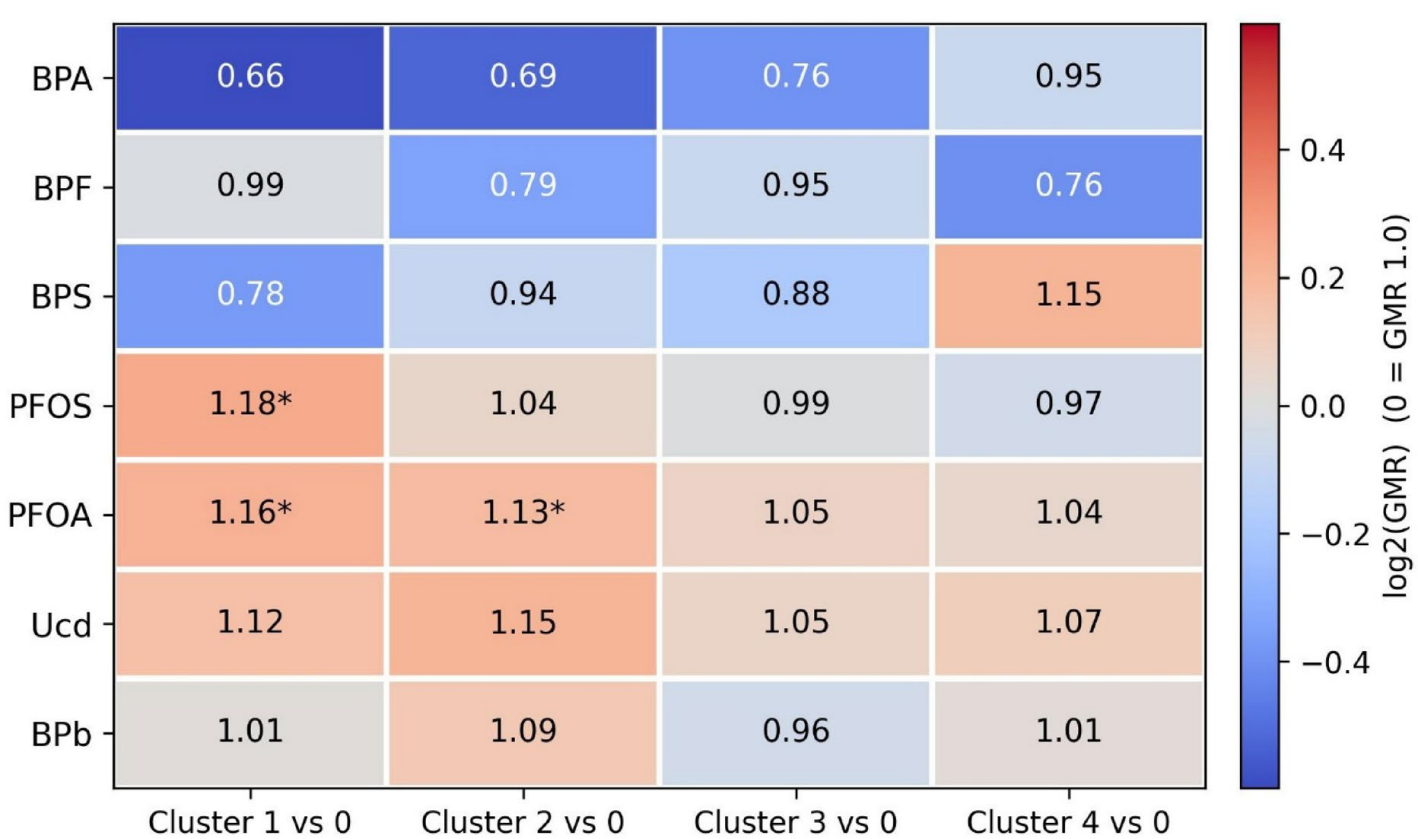
Summary heatmap of adjusted geometric mean ratios (GMRs) across dietary-behavioral clusters after false discovery rate (FDR) correction. This heatmap provides a visual summary of adjusted GMRs comparing each dietary-behavioral cluster with the reference cluster (Cluster 0). Colors represent log₂-transformed GMR values centered at zero (GMR = 1.0), allowing symmetric visualization of the direction and magnitude of exposure differences across clusters. Numeric labels indicate the original GMR estimates. Statistically significant comparisons after FDR correction are indicated by asterisks (*FDR *p* < 0.05; **FDR *p* < 0.01; ***FDR *p* < 0.001). Models were adjusted for age, sex, and urinary cotinine; urinary cadmium was additionally adjusted for urinary creatinine. This figure summarizes the adjusted exposure differences that remained statistically significant after FDR correction. BPA: bisphenol A; BPF: bisphenol F; BPS: bisphenol S; PFOS: perfluorooctane sulfonate; PFOA: perfluorooctanoic acid; Ucd: urinary cadmium concentration; BPb: blood lead concentration.

In the omnibus test of the covariate-adjusted WLS model, PFOS (*p* = 0.0107), PFOA (*p* = 0.0085), BPb (*p* = 0.0075), and BPA (*p* = 0.0318) showed significant overall differences between clusters (Table 4). However, after false discovery rate (FDR) correction of pairwise comparisons, statistically significant between-cluster differences were retained only for selected PFOS and PFOA comparisons ( 5; Fig. 5). No significant cluster difference was observed in the omnibus test for BPF (*p* = 0.0940), BPS (*p* = 0.0733), or Ucd (*p* = 0.4147) (Table 4). In adjusted GMR comparisons using Cluster 0 as the reference, PFOS was significantly higher in Cluster 1 (GMR, 1.182; 95% CI, 1.052–1.328; FDR *p* = 0.0478). PFOA was also significantly higher in Cluster 1 (GMR, 1.161; 95% CI, 1.046–1.290; FDR *p* = 0.0478) and Cluster 2 (GMR, 1.131; 95% CI, 1.046–1.223; FDR *p* = 0.0478) (Tables 4 and 5; Figs. 4 and 5). Blood lead showed a directionally higher level in Cluster 2 than in Cluster 0 (GMR, 1.091; 95% CI, 1.013–1.176), but this comparison did not remain statistically significant after FDR correction (FDR *p* = 0.1037) (Tables 4 and 5; Fig. 5). No bisphenol comparison remained statistically significant after FDR correction, and Ucd showed no significant cluster difference after covariate adjustment (Tables 4 and 5; Fig. 5). Taken together, the adjusted analysis showed that the most robust between-cluster differences were observed for PFAS biomarkers. Higher PFOS and PFOA concentrations were observed in Cluster 1, characterized by a basic-food and seafood-oriented profile, whereas higher PFOA was observed in Cluster 2, characterized by a packaging/contact-oriented profile (Tables 2 and 4, and 5; Fig. 5). Blood lead in Cluster 2 showed an elevated direction, but this pattern was not statistically significant after FDR correction in the adjusted model including sex (Tables 4 and 5; Fig. 5).

**Table 4:** Adjusted geometric mean ratios of chemical biomarkers by dietary-behavioral cluster.

Exposure	C1 vs. C0	C2 vs. C0	C3 vs. C0	C4 vs. C0	Omnibus *p*
BPA	0.661 (0.474, 0.923)	0.694 (0.520, 0.928)	0.764 (0.580, 1.008)	0.948 (0.683, 1.315)	0.0318
BPF	0.989 (0.738, 1.325)	0.788 (0.619, 1.004)	0.947 (0.743, 1.207)	0.755 (0.579, 0.986)	0.0940
BPS	0.777 (0.604, 0.999)	0.939 (0.737, 1.198)	0.879 (0.702, 1.101)	1.153 (0.866, 1.535)	0.0733
PFOS	1.182 (1.052, 1.328)	1.042 (0.962, 1.129)	0.995 (0.911, 1.087)	0.966 (0.876, 1.065)	0.0107
PFOA	1.161 (1.046, 1.290)	1.131 (1.046, 1.223)	1.054 (0.974, 1.139)	1.038 (0.951, 1.133)	0.0085
Ucd	1.121 (0.955, 1.317)	1.154 (0.971, 1.371)	1.049 (0.900, 1.224)	1.072 (0.879, 1.309)	0.4147
BPb	1.007 (0.936, 1.084)	1.091 (1.013, 1.176)	0.964 (0.896, 1.038)	1.015 (0.944, 1.092)	0.0075

Table 4 presents adjusted geometric mean ratios (GMRs) and 95% confidence intervals (CIs) of chemical biomarker concentrations comparing dietary-behavioral clusters identified using UMAP-based K-means clustering, with Cluster 0 as the reference group. Estimates were obtained from weighted least squares regression models applied to natural log-transformed biomarker concentrations. The models were adjusted for age, sex, and urinary cotinine; urinary creatinine was additionally included for urinary cadmium to account for urinary dilution. Omnibus p values indicate the overall cluster effect for each biomarker. FDR-adjusted p values for pairwise comparisons are presented in Table 5. BPA: bisphenol A; BPF: bisphenol F; BPS: bisphenol S; PFOS: perfluorooctane sulfonate; PFOA: perfluorooctanoic acid; Ucd: urinary cadmium concentration; BPb: blood lead concentration.

**Table 5:** FDR-adjusted p values for pairwise adjusted GMR comparisons.

Exposure	C1 vs. C0	C2 vs. C0	C3 vs. C0	C4 vs. C0
BPA	0.0839	0.0839	0.1597	0.8372
BPF	0.9413	0.1597	0.7964	0.1571
BPS	0.1597	0.7814	0.5257	0.5447
PFOS	0.0478*	0.5447	0.9413	0.6885
PFOA	0.0478*	0.0478*	0.4143	0.6331
Ucd	0.3794	0.2674	0.7185	0.6885
BPb	0.9121	0.1037	0.5447	0.7964

Table 5 presents Benjamini–Hochberg false discovery rate (FDR)-adjusted p values for the pairwise adjusted GMR comparisons shown in Table 4. The adjusted models included age, sex, and urinary cotinine; urinary creatinine was additionally included for urinary cadmium. Asterisks indicate comparisons that remained statistically significant after FDR correction. *FDR *p* < 0.05. GMR: geometric mean ratio; FDR: false discovery rate; BPA: bisphenol A; BPF: bisphenol F; BPS: bisphenol S; PFOS: perfluorooctane sulfonate; PFOA: perfluorooctanoic acid; Ucd: urinary cadmium concentration; BPb: blood lead concentration.

## Discussion

In this study, diet- and nutrition-related behaviors were integrated into multi-item dietary-behavioral profiles, clusters were derived in a latent space, and PFAS, bisphenol, and metal biomarker concentrations were compared across clusters. The main findings were that PFOS and PFOA concentrations were higher in the basic-food and seafood-oriented cluster, whereas PFOA was also higher in the food packaging/contact-oriented cluster. In the adjusted model including sex, PFOS was higher in Cluster 1 than in Cluster 0 (GMR = 1.182; 95% CI, 1.052–1.328; FDR *p* = 0.0478), and PFOA was higher in Cluster 1 (GMR = 1.161; 95% CI, 1.046–1.290; FDR *p* = 0.0478) and Cluster 2 (GMR = 1.131; 95% CI, 1.046–1.223; FDR *p* = 0.0478) (Tables 4 and 5; Figs. 4 and 5). Blood lead (BPb) in the packaging/contact-oriented cluster showed an elevated direction, but this comparison did not remain statistically significant after FDR correction in the adjusted model including sex. In contrast, cluster differences in bisphenols and urinary cadmium (Ucd) were not robust after considering covariates and multiple comparisons. These findings suggest that not only dietary composition itself but also behavioral contexts, such as packaging and food-container contact, may be related to group-level differences in environmental chemical exposure. In particular, the PFAS findings are consistent with recent risk assessment perspectives emphasizing food intake as an important contributor to internal PFAS burden and the need for long-term exposure management by identifying potentially vulnerable groups^24^.

In the case of bisphenols, a single urine-based measurement can be substantially affected by temporal variability, which can limit time alignment with cluster-based behavioral indicators. Previous reports have shown that urinary BPA varies greatly depending on the sampling method (spot, first morning, 24-hour) and daily fluctuations, which may explain why between-cluster differences can be attenuated even when a “packaging and contact propensity” is present^25^. In this study, BPA showed some pairwise differences before FDR correction, but no bisphenol comparison remained significant after FDR correction (Table 5). In addition, approaches that systematically address uncertainty in exposure assessment, including measurement error and limitations of proxy indicators, provide an important background for interpreting the attenuated bisphenol results after FDR correction in this study^26^. For blood lead, exposure may reflect multiple chronic sources, including environmental, residential, and occupational factors, and its association with cardiovascular risk has been continuously examined^27^. However, in the present analysis, the BPb elevation in Cluster 2 did not remain significant after FDR correction in the adjusted model including sex, and therefore should be interpreted as a suggestive pattern rather than a robust cluster difference (Table 5). Furthermore, literature that comprehensively discusses environmental toxicity threats from the perspective of vulnerability, including child development, supports the public health need to design preventive strategies by identifying vulnerable exposure groups^28^.

Substance- and matrix-specific characteristics are important when interpreting metal results. Although cadmium reflects accumulation and long-term exposure in the body, urinary concentration may be interpreted differently depending on kidney accumulation, excretion dynamics, and urinary dilution correction methods. In fact, a study that empirically deals with the relationship between kidney and urinary cadmium suggests that Ucd may reflect accumulation and kidney functional characteristics together rather than only “current exposure”^29^. In addition, it has been emphasized that although creatinine correction is essential in urinary biomarker analysis, the results may vary because the creatinine distribution itself is linked to age, sex, and muscle mass at the population level^30^. Therefore, the weakening of the cluster difference in Ucd after adjustment in this study suggests the possibility that urinary dilution, covariate structure, and the complexity of long-term cumulative indicators contributed more strongly than the diet- and behavior-related differences captured by the cluster^29,30^. This interpretation is in line with the research direction raised by the exposome concept dealing with “the overall structure of exposure,” and repeated measurements and multilayer exposure integration are needed in the future^31^.

The public health implication of this study is to show how dietary and behavioral structures may organize environmental exposure profiles before directly linking dietary patterns to disease. In particular, attempts to quantify the disease burden of foodborne exposure in the global food safety and risk assessment framework support that behavior-based clusters can be used for risk communication and policy design^32^. Indeed, reports that bisphenols and toxic elements can be detected simultaneously in ready-to-eat foods remind us that the use of packaged and processed foods can be a potential context for combined exposure^33^. In addition, a study that analyzed the risk of metabolic syndrome by machine learning by considering both nutritional intake and environmental factors shows that the “unsupervised mapping + exposure biomarker comparison” approach in this study is aligned with the data-driven direction within nutritional epidemiology^34^.

In the case of PFAS, human exposure through food is an important route, and biomonitoring and risk assessment can be used as the basis for exposure reduction strategies^35^. In particular, a review that systematically deals with food contributions provides a background for explaining that seafood consumption can be linked to PFAS internal burdens in some groups^36^. As health effects and exposure evidence are widely accumulated for bisphenols, even if the significance is weakened in this study, the importance of the exposure route itself does not disappear^37^. The epidemiological basis for the health effects of PFAS may be heterogeneous by substance and indicator, but it emphasizes the need for monitoring in terms of continuous exposure and long-term internal burden^38^. In addition, the evidence that phenol exposure, including BPA, is observed at the population level supports that packaging and contact behavior is meaningful over a wide range^39^. Finally, the latest review, which summarizes the association of specific dietary patterns with health indicators, shows that a pattern-based approach is gradually becoming standardized, suggesting the possibility of expanding research explaining health differences by integrating dietary patterns and environmental exposure in the future^40^.

Several limitations should be considered when interpreting these findings. First, this study used a cross-sectional secondary analysis design, and therefore the results should not be interpreted as causal evidence linking dietary-behavioral profiles to exposure sources or disease outcomes. Second, the dietary-behavioral clusters were derived from questionnaire-based proxy indicators, which may not fully capture actual intake amounts, specific food brands, cooking materials, or packaging characteristics. Third, biomarker time windows differed across chemical groups. Serum PFAS reflect relatively persistent internal burdens, whereas urinary bisphenols may be affected by spot-sample timing and short-term variability, and urinary cadmium may be influenced by urinary dilution and kidney-related excretion dynamics. Fourth, although survey weights were used in descriptive and regression-based comparisons, the unsupervised clustering procedure itself did not fully incorporate all components of the complex survey design. Finally, residual confounding by occupation, residential environment, water contamination, historical exposure, and unmeasured lifestyle factors cannot be excluded. Future studies should validate these profiles using repeated biomarker measurements, more detailed dietary and packaging information, and longitudinal designs.

## Conclusion

In this study, unsupervised learning from KoNEHS adult data was used to map diet- and nutrition-related behaviors into a latent space, derive clusters, and compare environmental chemical biomarker concentrations by cluster. PFOS and PFOA were higher in the basic-food and seafood-oriented cluster, and PFOA was also higher in the packaging/contact-oriented cluster after covariate adjustment and FDR correction. Blood lead in the packaging/contact-oriented cluster showed an elevated direction, but this pattern did not remain statistically significant after FDR correction in the adjusted model. These findings suggest that behavioral contexts such as packaging and food-container contact, as well as dietary composition, may help structure group-level differences in environmental chemical exposure profiles. However, because this study used cross-sectional secondary survey data, the findings should be interpreted as exploratory population-level exposure patterns rather than causal evidence of specific exposure sources.

## Data Availability

The data analyzed in this study were obtained from the Korean National Environmental Health Survey (KoNEHS), conducted by the National Institute of Environmental Research (NIER), Republic of Korea. The KoNEHS raw datasets are not redistributed by the authors because of data protection regulations, but they can be accessed through the official data application and approval process of the National Institute of Environmental Research. Information on data access and application procedures is available through the Environmental Health Information System data portal (https://www.ehtis.or.kr/cmn/sym/mnu/mpm/62002000/htmlMenuView.do).The analytical code used for data preprocessing, UMAP-based clustering, weighted geometric mean estimation, adjusted GMR analysis, false discovery rate correction, and table/figure generation has been deposited in Zenodo: https://doi.org/10.5281/zenodo.21365510. The Zenodo repository does not include KoNEHS raw data or individual-level derived data. Users must obtain access to the KoNEHS raw data through the official NIER data application process to reproduce the analyses.
